# Engagement during a Mixed In-Person and Remotely Delivered Psychological Intervention for Youth with Functional Abdominal Pain Disorders and Anxiety

**DOI:** 10.3390/children8090775

**Published:** 2021-09-02

**Authors:** Alaina K. Miller, Samantha L. Ely, Brittany N. Barber Garcia, Patricia Richardson, Natoshia R. Cunningham

**Affiliations:** 1 Department of Psychology, Wright State University, Dayton, OH 45435, USA; miller.1556@wright.edu; 2 Department of Family Medicine, Michigan State University, Grand Rapids, MI 49503, USA; elysaman@msu.edu; 3 College of Human Medicine, Michigan State University, Grand Rapids, MI 49503, USA; Brittany.BarberGarcia@helendevoschildrens.org (B.N.B.G.); patricia.richardson@spectrumhealth.org (P.R.); 4 Helen DeVos Children’s Hospital (Spectrum Health), Grand Rapids, MI 49503, USA

**Keywords:** functional abdominal pain, anxiety, pain, telehealth, mhealth, internet intervention, cognitive behavioral therapy

## Abstract

Functional abdominal pain disorders (FAPD) are common disabling pain conditions frequently associated with co-occurring mental health problems such as anxiety. Psychological therapies such as cognitive behavioral therapy (CBT) have been shown to be effective. Therefore, it is important to understand participant engagement (i.e., use of intervention tools) to such approaches, and if engagement impacts treatment response. The Aim to Decrease Anxiety and Pain Treatment (ADAPT) is an effective psychological treatment approach using a blend of in-person sessions and interventionist phone support with self-paced web modules to manage pain and anxiety. The current study used a mixed-methods approach to investigate micro-level and macro-level participant engagement during the ADAPT program. In-person/phone session attendance was high (>95%) although scheduling adjustments were common (25.5%). Varied levels of engagement with web tools were observed. Thematic analysis also revealed variability in patterns of use. Additionally, while participants indicated they generally understood how to use certain skills (e.g., problem solving, detective thinking), and these skills were effective in managing symptoms during treatment, these activities were generally underutilized. Further, participant engagement did not predict response to the ADAPT intervention. These findings are important as the demand for accessible psychotherapeutic tools to manage pain and anxiety is likely to remain high.

## 1. Introduction

Functional abdominal pain disorders (FAPD), a chronic recurrent set of pain conditions, are common among children and youth worldwide [1,2,3]. While rates vary, pooled data suggests FAPD affects nearly 20% of youth in the US [3] and globally [2]. A large portion (30%) of affected youth continue to experience symptoms for five years or longer [4]. Anxiety, or fears or worries, that cause impairment in functioning [5], are also common in youth with FAPD (impacting between 40% to 67% [6] of youth) [6,7,8,9] and are associated with more severe pain symptoms and increased pain-related impairment [4,6,8,10,11] over the long term [10,11]. For multiple reasons the COVID-19 pandemic has likely further impacted emotional functioning [12] of these already vulnerable youth. Indeed, the prevalence rates of anxiety and depression in children have increased during the pandemic [13]. Therefore, effective and accessible approaches to address symptoms are critical.

A cognitive behavioral therapy (CBT) approach incorporating cognitive and behavioral [14] strategies can be effective for youth with chronic pain [15], including FAPD [15,16,17,18], with evidence of reduction in pain-related impairment maintained over a period of one year or longer [4]. Further, CBT is considered the gold standard treatment for pediatric anxiety [19,20]. One evidence-based approach, the Aim to Decrease Abdominal Pain Treatment (ADAPT) [21,22], is a 6-session weekly individual treatment (2 in-person sessions and 4 web-based sessions with interventionist phone support) incorporating CBT strategies from pain management [23] and anxiety reduction [24] protocols to treat FAPD and co-occurring anxiety. ADAPT has been shown to significantly reduce disability due to pain and anxiety symptoms in youth with FAPD in a randomized clinical trial (RCT) [22]. A moderate effect on pain symptoms was also observed. Remote psychological interventions with a therapist-patient dyad and access to self-paced web tools in ADAPT has been shown to be feasible while increasing accessibility [21]. However, youth adherence to intervention/treatment regimens and particularly web tools is generally variable [25], and this was also observed in ADAPT [22]. Other programs with web-based and telehealth components to manage pediatric pain also demonstrate variable adherence (e.g., 30% completion rate [26]); yet engagement to such interventions is poorly understood [27].

Engagement, defined here as use of intervention tools that lead to health behavior change [28], is a method for understanding use of web-based tools that are part of a behavioral health intervention. Engagement is further conceptualized at the micro-level as the extent of intervention use by the participant as well as user experience (e.g., amount, duration, breadth, and depth of usage), and at the macro-level as participant behavior change (e.g., effectiveness of tool itself) [28]. There is evidence that poor patient engagement minimizes response to a pediatric CBT intervention [27]. While there are some data on adherence to ADAPT [21,22], (which may be conceptualized as an aspect of micro-level engagement), youths’ broader engagement with ADAPT, including macro-level factors, are less well understood. More data are needed to better understand engagement to such treatment approaches, and how engagement is related to their effectiveness.

The study objective is to examine engagement via participant use of ADAPT through a mixed methods approach. Using data pooled from prior investigations of ADAPT [21,22,29], we examine use of in-person and remotely-delivered portions (e.g., interventionist phone calls and web tool use) of the ADAPT intervention. We explore youth engagement with the ADAPT web tools via quantitative (e.g., number and types of web tools used) and qualitative (e.g., thematic analysis by experts to explore patterns of web tool use) methods. We also examine if participants’ use of web tools were effective during the intervention. Finally, we explored whether youth who demonstrate higher levels engagement (e.g., higher rates of web tool use) improve more (e.g., post-treatment reductions in pain-related disability, anxiety, functional impairment) after ADAPT.

## 2. Methods

### 2.1. Procedure and Sample

**Figure 1:**
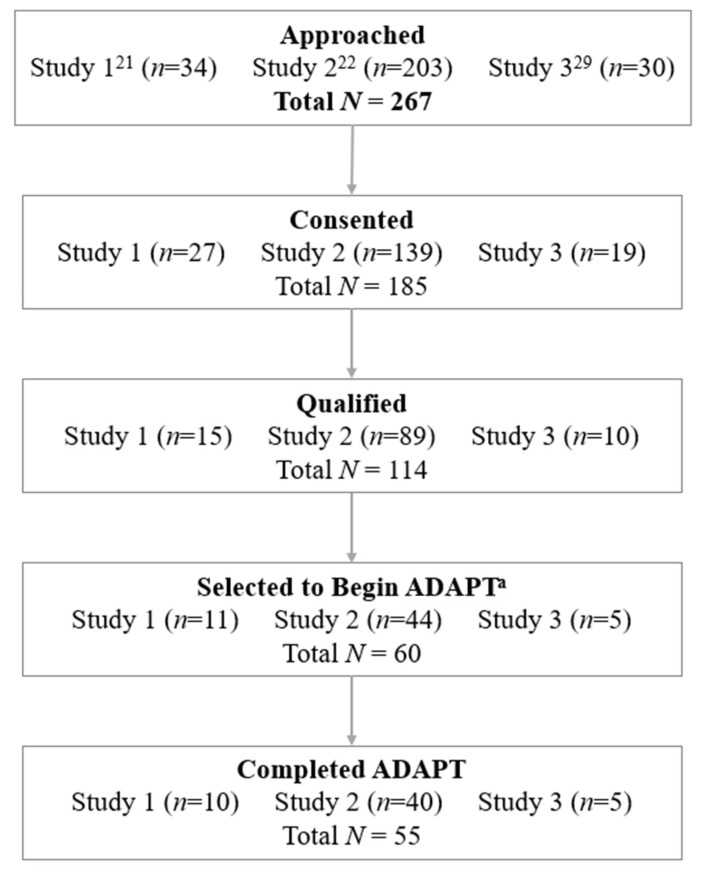
Participant flow. Potentially eligible patients were approached to participate. Consenting participants were assessed for study eligibility. Of those, a portion of qualifying participants were then selected to begin ADAPT. Of the 60 total participants selected to begin ADAPT, 57 began the program and 55 completed the ADAPT protocol. For more detail on eligibility criteria and exclusion of participants, please see the original studies [21,22,29]. *Note*: ^a^ All participants were offered ADAPT (Study 1) or were randomized to ADAPT (Studies 2 & 3).

The current investigation is a secondary analysis of data from several studies that utilized ADAPT for the treatment of pain-related symptoms and anxiety for youth with FAPD (see Figure 1). Data were used from the following investigations: (1) A pilot/development study [21] aimed to examine the feasibility and acceptability of ADAPT, (2) A RCT exploring the effect of ADAPT on disability, pain, and anxiety as compared with standard medical care [22], and (3) a study aimed to examine the changes in neural mechanisms of pain after undergoing ADAPT (clinicaltrials.gov NCT03518216) [29].

Participants and their caregivers were recruited from outpatient pediatric gastroenterology clinics at an academic medical center.

Across all studies, participants with a diagnosis of a functional abdominal pain disorder were eligible to qualify. They and their caregiver had to be English speaking with no evidence of developmental disabilities or cognitive delays. Participants were ineligible if they had evidence of another organic medical condition with abdominal pain as part of the presenting complaint (e.g., Inflammatory Bowel Diseases), or if they were actively participating in psychotherapy for anxiety or pain.

There were a few minor differences in enrollment criteria across studies. While the age range was similar, as all participants were between ages 9 and 16 years, the criteria differed slightly (e.g., 9–14 years for two investigations [21,22]; and 11–16 years for the other investigation [29]). In addition, those in the neuroimaging study had to be MRI compatible (e.g., no braces or implant devices [29]). All three studies required participants to endorse more than minimal disability as evidenced by the FDI, and two of the three studies [21,29] also required elevations in anxiety and pain levels. For further detail on the studies’ methodologies, please refer to the original investigations [21,22,29].

### 2.2. ADAPT Intervention

ADAPT is a brief, evidence-based cognitive behavioral intervention to treat youth with co-occurring abdominal pain and anxiety [21,22]. It is generally comprised of 6 child-focused individual sessions with an interventionist (2 in-person sessions and 4 self-paced web sessions with interventionist phone support). Content included cognitive (e.g., problem solving, detective thinking [in-depth cognitive restructuring]) and behavioral (e.g., stepladders [gradual exposure], relaxation training) strategies to manage pain and/or anxiety. See Table 1 for a detailed outline of the program protocol.

Web modules were accessible via an online portal that participants completed in a self-paced manner prior to scheduled phone calls with an interventionist. Each web module included a blend of passive (e.g., watching instructional and therapist/patient videos demonstrating skill use, viewing handouts), and interactive (e.g., completing activities and forms) tools for participants to complete each week. Videos were generally watched prior to completing interactive activities; handouts could be downloaded on a side panel at any point during the module.

### 2.3. Outcome Measures

Participants also completed measures on pain- and anxiety-related outcomes before and after undergoing the ADAPT intervention.

The Functional Disability Inventory (FDI), a 15-item measure, was used to assess pain-related disability on a scale of 0–60 [30]. Higher scores indicate a greater level of disability, with clinical cut-offs for mild, moderate, and severe disability [31]. This scale demonstrated high reliability (α = 0.88).

Pain intensity via a visual analog scale (VAS) was used to measure average pain intensity over the past two weeks, with scores ranging from 0 (no pain) to 10 (worst pain) [32].

The Screen for Child Anxiety-Related Disorders (SCARED) measured participant reported anxiety symptoms on a scale from 0–82, with higher scores indicating greater anxiety. The SCARED has an established cut-score of ≥25 to indicate clinically significant anxiety [33]. This scale demonstrated excellent internal reliability (α = 0.95).

### 2.4. Data Analysis

After evaluating sample characteristics, a blend of qualitative and quantitative data analytic approaches detailed below were used to understand micro-level (e.g., phone attendance, use of web tools) and macro-level (e.g., effect of specific skills) engagement to the ADAPT program. Engagement in relation to clinical outcomes was also explored.

#### 2.4.1. Micro-Level Engagement

##### Quantitative Methods

Session attendance was calculated by examining the proportion of youth completing in-person and virtual/phone sessions. Information regarding scheduling re-adjustments (e.g., cancellations, rescheduling, and combined sessions) was also collected. Use of ADAPT web tools was also examined drawing from methods to calculate treatment adherence as detailed in our prior research [21]. Rates of overall use (a total percentage score, then categorized into low, moderate, and high usage), as well as use of specific kinds of web tools (e.g., videos, handouts, and forms) were calculated by taking the proportion of items used by the participant to the total number of items available to the participant. In addition, we investigated the specific use of passive and interactive tools. Tools were separated by type of use given the literature showing differences between active and passive coping strategies in relation to outcomes of youth with functional abdominal pain conditions [34,35]. Percent of passive (e.g., videos watched, handouts downloaded) and interactive (e.g., forms completed) web tools used were calculated.

For interactive skills (problem solving, detective thinking, stepladders) that required participants to write in responses, appropriate use was examined by recording whether or not the participant generally responded to prompts as intended on a dichotomous (“1,” generally used appropriately versus. “0,” not used appropriately) scale. Effect of skills (problem solving and detective thinking; see macro-level engagement section) was also assessed in a similar manner.

##### Qualitative Methods

Drawing from grounded theory approach [36], used in the principal investigator’s (NRC) prior research [21,37,38], qualitative thematic analysis was used to better understand themes and patterns of ADAPT web tool use. Qualitative analytic methods employed allowed for visual observation of patterns that emerged in the aggregated data. This inspection of data led to observation of trends and themes, which allowed for quantification via traditional statistical analyses to provide a rich and comprehensive approach to understanding ADAPT web tool use.

The qualitative analytic strategy involved a binning and winnowing process used in previous pediatric pain research [38]. This method allowed for identification, categorization, and coding uses of ADAPT into “bins”. Bins were then winnowed by condensing redundant observations into thematically similar groups or domains. Prior to examination of the data, the research team identified several domains (e.g., patterns of use, appropriate use) to categorize micro-level engagement to ADAPT. ADAPT web tool usage data was reviewed by three independent coders: a psychologist (NRC), a psychology doctoral student (AKM), and a post-baccalaureate researcher (SLE).

Data was reviewed independently then discussed by the team to establish concordance for themes of use. Cross-checking between observers was employed to establish reliability.

#### 2.4.2. Macro-Level Engagement

##### Mixed Methods Approach

Macro-level engagement was also evaluated through exploring data gleaned from two tools within the web portion of the ADAPT intervention (problem solving and detective thinking) where participants were directly able to indicate how effective the strategy was for them in the moment. Self-report questions (e.g., “What happened when you tried the solution?” for problem solving; Worry rating 0–10 [with 0 indicating not at all and 10 indicating highest] before and after detective thinking) were used to assess effectiveness of skills taught.

#### 2.4.3. Engagement in Relation to ADAPT Outcomes

To confirm treatment effects in the pooled sample (e.g., pain-related disability, pain intensity, and anxiety) of ADAPT in the present sample, we conducted paired-samples *t*-tests. We also explored whether youth with higher levels engagement (e.g., web tool use) improved more in these outcomes (i.e., had greater post-treatment reductions) after undergoing ADAPT. Three separate MANCOVA models assessing post-treatment disability and pain levels and three ANCOVA models assessing anxiety were conducted to investigate treatment outcome differences between different aspects of engagement (e.g., low, moderate, and high use; active versus passive engagement; overall patterns of use). Each analysis investigated a different aspect of engagement and controlled for the respective pre-treatment scores and age.

## 3. Results

### 3.1. Sample Characteristics

The total sample consisted of 60 youth with abdominal pain ages 9–15 years (*M* = 11.7, *SD* = 1.7 years). The sample was primarily comprised of Caucasian youth (*n* = 50, 83.3%) and included 32 females (53.3%). A one-way ANOVA (age) and chi-square tests (race and gender) revealed no significant differences between participants recruited across studies (all *p*’s > 0.05). The overall sample was categorized by moderate levels of anxiety (*M* = 36.2, *SD* = 17.4), disability (*M* = 20.5, *SD* = 9.7), and pain (*M* = 4.0, *SD* = 1.9) and there were no significant differences in these characteristics across separate studies (all *p*’s > 0.05). In addition, no significant differences were shown in post-treatment outcomes (disability, pain, and anxiety) between the studies (all *p*’s > 0.05). Further, there were no differences between participants’ overall web tool use across the separate investigations (*F*(2,52) = 0.931, *p* = 0.401). Correlation analyses revealed no associations between the percentage of total web tool items used and all other study variables (e.g., age, pain levels, functional disability, anxiety; all *p’s* > 0.05). In addition, no sex differences in overall use of web tool items were noted (*t*(53) = 0.11, *p* = 0.912).

### 3.2. Micro-Level Engagement

#### 3.2.1. Quantitative Results

Session attendance. In total, 60 youth with abdominal pain were assigned to ADAPT (See Figure 1). Of those assigned, 57 (95%) started the program (3 withdrew at baseline). Then, only two participants failed to complete the program, defined as not completing the post assessment. Of those, one completed both in-person sessions and no web/phone sessions, and one completed one in-person session only before dropping out.

Of the 60 participants assigned to ADAPT, 55 (*n* = 91.7%) completed treatment. Of the 55 who completed treatment, nearly all (*n* = 54; 98.2%) youth attended all in-person sessions. Most ADAPT completers (*n* = 54; 98.2%) attended all required phone calls during the remotely delivered portion of the intervention. The remaining participant attended at least half (75%) of the required calls and missed the final call due to familial circumstances and time constraints.

Additional information about whether or not scheduling re-adjustments (e.g., cancellations, rescheduling, and combined sessions) were needed for those who completed ADAPT was also recorded. In total, 14 (25.5%) participants required a scheduling adjustment. Specifically, several participants (*n* = 6, 10.9%) requested combined sessions prior to beginning the ADAPT program. These were due to distance from the medical center and other scheduling conflicts. Eight (14.6%) ADAPT completers needed a scheduling readjustment after the program commenced (for either in-person or phone sessions with the interventionist) at least one time, with 2 completers (3.6%) needing 2 or more adjustments.

Rates and frequency of use of ADAPT web tools. On average, participants used approximately 31.4% of all web tools. To conceptualize degree of web tool use, participants were stratified into three categories (based on tertiles of overall use), ranging from low (0–23%), moderate (24–40%), and high use (41–73%). Seventeen (30.9%) participants were considered low users—seven of which (12.7% of total participants) did not complete any online content—18 (32.7%) were considered moderate users, and 20 (36.4%) were considered high users. Disclosed reasons for non-use of web tools were difficulties with internet access (*n* = 3, 5.5%) and website login (*n* = 2, 3.6%). Two (3.6%) participants did not disclose reasons for non-use of tools.

The percentage of available videos and handouts viewed, and forms completed was also calculated. Participants watched, on average, over one-third of the available videos (*M* = 41.9%, *SD* = 34%, range = 0–100%). On average, participants downloaded 22.9% (*SD* = 30.5%, range = 0–100%) of the handouts and completed 30.9% (*SD* = 20.6%, range = 0–64%) of the online forms. For a breakdown of component use by session/skill, please see Table 2.

Use of passive and interactive tools. Out of the 48 participants (87.3%) who used web tools, 45 participants (93.8%) used tools passively by either downloading handouts and/or watching videos (the video was the first prompt of the session and handouts were available at all times on a side bar), and 43 participants (89.5%) engaged with tools interactively by completing at least some skill practice forms. Five participants (10.4%) engaged passively only, 3 participants (6.3%) engaged interactively only, and the majority (40 participants; 83.3%) used a combination of passive and interactive components.

#### 3.2.2. Qualitative Results

We explored two domains of micro-level engagement: patterns of use and appropriateness of use. Within the patterns of use domain, consistency of use was identified as a theme. Consistency of use describes a participant’s longitudinal pattern of use across the ADAPT intervention period. Participants were coded and stratified into several categories based on these patterns. Appropriate use (whether or not it could be reasonably discerned that the participant understood how to use the skill) was another domain investigated in the free response sections.

Patterns of Use. Four patterns detailing consistency of use emerged: no use (not accessing any online content via videos, handouts, or forms), intermittent use (defined as inconsistent/varied use of web tools throughout the program), high initial use followed by no/limited use as the program progressed, and consistent use throughout the program. Seven (12.7%) participants did not use any web tools. Twenty-six (47.3%) participants used the platform intermittently. Ten participants (18.2%) started to use online web tools initially but stopped. Twelve (21.8%) participants used the web tools consistently (i.e., they accessed web content weekly).

Appropriate use of problem solving, detective thinking, and stepladders activities was also examined. The majority of the skills were used appropriately with some caveats. Of the 38 (69.1%) participants who completed problem solving, 33 (86.8%) used the overall skill appropriately. Of those who did not complete the skill appropriately, three (7.9%) left questions blank and two (5.3%) did not provide responses that matched the questions (e.g., indicated “belly pain” as a coping skill). Of note, 11 (28.9%) participants struggled to identify a problem (even if they went on to use the skill appropriately thereafter), but instead identified a feeling such as “Anxiety/stress” or “belly pain”. Furthermore, one specific question within the problem solving activity asked participants to identify thoughts, feelings, and behaviors in a single free response. Most (*n* = 31, 81.6%) did not identify all three (e.g., only labeled a thought and feeling, but not a behavior), even if they otherwise used the problem-solving skill appropriately. Other participants demonstrated a potential lack of understanding of the difference between thoughts, feelings, and behaviors, such as one participant noting, “I feel like doing nothing only laying down.”

Of the 26 (47.3%) participants who completed detective thinking, the majority (*n* = 22; 84.6%) used the skill appropriately. One (3.8%) participant left a portion of the activity blank, whereas three (11.5%) of participants did not fully address the questions asked. For example, one participant indicated, “[When] I can’t see my mom all the time,” as a new realistic thought when trying to overcome worries about a test.

Of the 15 (27.3%) participants who completed the stepladders activity, about half (*n* = 8; 53.3%) completed the skill appropriately. When asked to indicate a goal to overcome a fear, four (26.7%) participants did not indicate a fear, but rather a general goal such as, “[To] be a good student in school.” Further, six (40.0%) participants did not list sequential steps to overcome their fear, but rather indicated to-do lists or ideas on how to obtain a goal (e.g., “Do homework” followed by “Participate in class.”).

### 3.3. Macro-Level Engagement

Effectiveness of strategies during ADAPT was also explored using mixed methods. Two activities within the ADAPT web content, problem solving and detective thinking, prompted participants to report their level of effectiveness after use. Of the 48 participants who used those web tools, 43.8% (*n* = 21) reported on their effectiveness (See Table 3 for examples). All participants (*n* = 20) who completed the problem-solving skills indicated it was effective in reducing pain/anxiety symptoms. The majority of ADAPT web tools users did not complete detective thinking. Of those who did, most (10/12; 83.3%) reported it was effective in reducing anxiety symptoms, with an average 4.5-point reduction on their worry ratings.

### 3.4. Web Tool Usage in Relation to ADAPT Outcomes

Paired samples *t*-tests confirmed the pooled sample showed significant reductions in disability (*t*(53) = 6.118, *p* < 0.001), pain (*t*(53) = 4.508, *p* < 0.001) and anxiety (*t*(53) = 5.475, *p* < 0.001) after undergoing the ADAPT program. We further examined differences in post-treatment outcomes based on web tool usage level (e.g., low, moderate, and high usage; active versus passive use; consistency of use). A MANCOVA controlling for age, baseline disability, and average pain revealed no significant differences in post-treatment disability and pain between groups (*F*(4, 94) = 0.170, *p* = 0.953). In addition, the other two MANCOVA models did not reveal significant differences related to active versus passive use (*F*(6, 92) = 0.098, *p* = 0.996) or consistency of use (*F*(6, 92) = 0.153, *p* = 0.988). Similarly, no significant group differences were found when examining post-treatment anxiety in an ANCOVA controlling for baseline anxiety and age (*F*(2, 49) = 1.005, *p* = 0.374). Further, there were no differences related to active versus passive use (*F*(3, 48) = 0.292, *p* = 0.831) or consistency of use (*F*(3, 48) = 0.318, *p* = 0.812).

## 4. Discussion

ADAPT is an evidence-based psychological treatment known to improve outcomes in youth with FAPD [21,22]. While the feasibility and efficacy of ADAPT has been established, it was unknown how components of ADAPT were utilized and if participant engagement affected response to treatment. The present study investigated aspects of both micro- and macro-level engagement with the ADAPT intervention. Findings suggest that participants were highly engaged in portions of the ADAPT intervention that included live interactions with a psychological provider (whether in-person or remote). On the other hand, a large degree of variability in use of ADAPT web tools was reported. While prior work suggested videos watched (mean usage 57%), forms downloaded (mean usage 21%), and interactive activities completed (mean usage 29%) in ADAPT varied [22], this was the first study to examine patterns of use (e.g., consistency of use), appropriateness of use, and effectiveness of specific web tools. Clear patterns of use emerged, and large portions elected not to use certain web tools. Of those who did use tools, the majority seemed to appropriately comprehend materials. Interestingly, the degree of overall web tool use (as defined by low, moderate, and high use) did not predict response to the ADAPT treatment. These findings are important as the demand for psychological treatment approaches for pediatric pain and anxiety that include remote interactions with live providers and self-paced web support tools will only continue to grow in the wake of the COVID-19 pandemic [39,40].

Prior to the COVID-19 pandemic, there was already support for the use of telehealth to address a number of pediatric complaints such as chronic pain [15], obesity [41], and sleep problems [42], with therapist-patient rapport rated as comparable to in-person treatments [43]. However, in spite of evidence of effect of such programs, actual patient engagement to remotely delivered therapies is less well understood. This was one of the first studies to comprehensively examine patient engagement during an evidence-based psychological treatment for pediatric chronic pain using a blend of in-person and remotely delivered platforms. Importantly, the current study allowed for examination of aspects of micro-level (e.g., frequency of web tools use) and macro-level (e.g., response to skills practice) engagement.

In terms of micro-level engagement, very high rates (>95%) of both in-person and phone session attendance rates were observed during ADAPT. In fact, participation in in-person versus remote (phone) interventionist sessions was nearly identical, suggesting that either approach is acceptable to families, with potentially less barriers to care (e.g., due to distance to psychological care) when using the remotely delivered option. Of note, a quarter of participants did request scheduling adjustments for either in-person or phone sessions at some point during the intervention. Therefore, some provider flexibility (on par with what would be expected in clinical settings) is key to successfully executing the program and likely contributed to the high rates of adherence observed to this component of the program. Perhaps this is because communicating with a provider is aligned with more conventional elements of medical care versus accessing web tools in the absence of a provider. Furthermore, these findings may speak to the importance of non-specific therapeutic factors in treatment [44], such as therapeutic alliance during a psychological intervention for youth. Indeed, the live interactions appeared to be the key element of the ADAPT treatment that was almost universally used regardless of degree of web tool use. Given this, nearly all participants could be considered moderately engaged based on high attendance rates to components with a live provider (whether in-person or remote). Program content was described and reviewed during these live sessions by the interventionist regardless of participant engagement (or lack thereof) in the self-paced web modules. These findings suggest solely web-based psychological treatments may limit the uptake and use of such strategies in the absence of live interaction with a psychological provider.

Interestingly, other research suggests comparably low levels of use (e.g., 30%) in other web-based pain management approaches [26,45]. In addition, the self-directed nature of technology-based interventions may lend way to variable engagement or even dropout, although such approaches are generally viewed positively by caregivers and youth [46,47]. Emerging research suggests novel and innovative technologies combined with positive reinforcement (e.g., use of gamification) are intended to increase engagement and may lead to behavioral health changes [48]. Examination of individual patterns of ADAPT web tool use showed variability among participants. For example, one third of participants could be categorized as low, moderate, or high overall users of web tools. Both interactive and passive tools were commonly and frequently used together, though some preferences for different types of web tools emerged for individual participants. By design, CBT focuses on teaching active coping strategies for pain management, and passive versus active coping strategies are associated with poorer outcomes in youth with FAPD [34,35]. Furthermore, interactive techniques (such as playing a video game) have been shown to positively impact pain related outcomes in youth [49]; thus, it is plausible that more interactive (versus passive) methods of engaging youth may enhance outcomes, although such effects were not observed in the current study.

Qualitative investigation into patterns of web tool use also revealed differences in participants’ consistency of use (e.g., consistent versus intermittent versus drop off versus no use at all) over time. Future examination into predictors and outcomes of different patterns of web tools use over time would be informative. Such approaches might be combined with other metrics of risk status in pediatric chronic pain samples [11,50].

When examining appropriate use of interactive activities, it is noteworthy the most common tools used were problem solving (69.1%), followed by detective thinking (47.3%), then stepladders (27.3%). Anecdotally, as skills became more complex, participant usage decreased. While problem solving and detective thinking were both understood by greater than 80% of users, stepladders (arguably the most complex skill taught) also had the lowest rates of appropriate use, with only about half using the skill correctly. Further, even for skills that were generally used appropriately, direct interventionist support for certain aspects of the skill (e.g., appropriately identifying thoughts, feelings, and behaviors) might be beneficial. Alternatively, structuring free response forms differently (e.g., include separate response fields for thoughts, feelings, and behaviors versus a single response field to address all three) may enhance the utilization of these skills. However, for complex and multi-faceted coping strategies such as gradual exposure, these activities may be most successfully executed with the support of an experienced provider.

Investigation into macro-level engagement showed that while specific tools (e.g., problem solving, detective thinking) to measure the effect of skills during the program were infrequently used, they were generally reported to be helpful when utilized. However, it is plausible those likely to use the tools may also be more likely to perceive the tools as effective possibly due to confirmation bias. It should also be noted these interactive skills were introduced at the end of the session when participants could have been more fatigued, these strategies generally require higher level of cognitive engagement, and tend to require more practice. Therefore, these factors should be taken into consideration when interpreting the results.

Of note, all the ADAPT web tools were designed for participants to use at their own pace, therefore participants may have only used tools that they felt comfortable completing or wanted more practice completing. Overall, the variable rates of web tool use as compared to the high rate of use of live interventionist support may suggest that participants perceived these web tools as complementary to the interventionist sessions rather than necessary.

Importantly, further investigation into the impact of engagement on participant outcomes revealed that web tool use and other engagement metrics (e.g., consistency of use) did not impact response to ADAPT across pain-related (functional disability and average VAS pain levels) and mental health-related (anxiety) outcomes. There is a robust psychotherapy literature acknowledging provider rapport as a key factor in psychology outcomes [44]. Further, there is sound evidence that in-person CBT interventions can impact functional and pain-related outcomes in youth with chronic pain [51]. Given the nature of ADAPT treatment is a hybrid model, the patterns of use could relate to participants’ views on the relative importance of the digital vs in-person content. Although the modest (~30%) rates of web tool use in ADAPT were comparable to those observed in other pediatric web-based interventions [26], it is unknown whether participants would have engaged with digital content differently if they were stratified into hybrid or digital only groups.

Interestingly, a systemic review [45] of digital interventions to treat anxiety and depressive symptoms in youth concluded that treatment effects were only seen in hybrid models where there was also therapist support. Interventions relying only on digital engagement alone did not observe clinically detectable improvements in treatment outcomes [45]. Arguably, it is possible that ADAPT led to improvements in clinical outcomes due to the *combined* therapist/web tool treatment delivery approach. Increased support from the provider in a hybrid live/web-based treatment approach may positively impact user engagement (e.g., sending reminders, reviewing completed materials). Cumulatively, this evidence suggests the adjunctive (versus critical) role of the web tools to support work with a skilled interventionist in a hybrid model of care.

A strength of the present investigation was the use of a mixed-methods approach [36,52,53] to understand micro-level and macro-level participant engagement to an evidence-based treatment. Through quantitative and qualitative methods, a comprehensive and robust understanding of the use of these tools is provided. Independent reviewers identified themes of use and established consensus in the few instances of conflicting reports. When patterns of web tool use were thematically observed (e.g., consistent use versus no use versus drop off versus intermittent use), these qualitative themes were then quantified by the investigative team to enrich understanding [52,53].

An additional strength of this investigation was the ability to simultaneously examine the micro-level and macro-level engagement factors. Further, the finding that web tool use, an aspect of micro-level engagement, was not associated with changes in pain-related disability, pain levels, or anxiety after a hybrid in-person/remotely delivered pain-focused intervention is a novel and relevant contribution to the literature.

This study has several limitations, including a modest sample size increased by pooling data from multiple investigations. This investigation also consisted of a predominantly Caucasian sample undergoing a single type of pain-focused psychological intervention. This limits generalizability to other racial and ethnic populations, age ranges, physical health problems, and mental health conditions. It would also be beneficial to examine the impact of caregiver engagement. ADAPT is predominantly child-focused though caregivers do play a role and presumably undertake responsibilities for scheduling/arranging provider visits. Caregiver perspectives including potential barriers to care, were not explicitly queried in this study but would be valuable for future investigations. Moreover, it should be noted ADAPT web tools were developed on a relatively modest budget whereas testing more technologically sophisticated (e.g., gamification, digital avatars, AI technologies) approaches specifically designed to optimize user engagement, and in some case, simulate a relationship with a real live provider, would be informative. Future research with more in-depth exploration of age and sex differences in tool use and impact on treatment outcomes is also needed.

This research was conducted prior to the COVID-19 pandemic, therefore access and availability of web-based tools in mental health care has been significantly impacted [54]. While access to psychological providers via telehealth [55], improved insurance coverage of virtual care [56], and patient acceptability of virtual psychological treatments has exponentially increased [57,58], this should not be conflated with provider access, which is still a major barrier to care. However, the increase in opportunities to access virtual care supports the importance of distilling and understanding patient engagement to such approaches.

In summary, understanding engagement with a psychological treatment for pediatric pain that includes a blend of live interventionist support and self-paced web-based modules provides useful information to providers as demands for telehealth and accessible psychological interventions are likely to increase.

## Figures and Tables

**Table 1:** ADAPT Protocol.

Session	Platform *	Skill/Module	Target
1	In person	Psychoeducation	Pain & Anxiety
		Relaxation: Deep Breathing & Guided Imagery	Pain
2	In person	Progressive Muscle Relaxation	Pain
		Calming Statements	Pain
		Activity Pacing	Pain
3	Web & phone	Pleasant Activity Scheduling	Pain
		Problem Solving	Pain
4 ^†^	Web & phone	Detective Thinking	Anxiety
5 ^†^	Web & phone	Stepladders	Anxiety
		Assertiveness Training	Anxiety
6	Web & phone	Maintenance Planning	Pain & Anxiety

* All sessions were generally 45–60 min per week with some variability. ^†^ For a small subset (*n* = 10), these sessions were excluded due to lack of clinically significant anxiety. Participants received a score on percent of tools completed based on what was assigned for each individual. Please refer to original clinical trial [22] for additional details.

**Table 2:** Web tool use of ADAPT skills.

	Passive Use				Interactive Use	
	Videos		Handout		Forms	
Skill Name	M (SD)	Range	M (SD)	Range	M (SD)	Range
Overall (*n* = 55)						
Pleasant Activities & Problem Solving	43.0% (39.4%)	0–100%	29.6% (40.6%)	0–100%	32.3% (22.9%)	0–50%
Detective Thinking	43.3% (43.4%)	0–100%	21.1% (36.1%)	0–100%	29.6% (28.6%)	0–67%
Stepladders	43.3% (44.7%)	0–100%	13.9% (31.8%)	0–100%	33.3% (47.7%)	0–100%
Assertiveness Training	38.9% (43.8%)	0–100%	13.3% (34.4%)	0–100%	28.2% (27.5%)	0–67%
Maintenance Planning	37.3% (45.4%)	0–100%	26.4% (43.9%)	0–100%	N/A	N/A

ADAPT is comprised of two in-person sessions and four therapist support calls with accompanying web modules. Web modules included videos, handouts, and interactive forms to deliver lesson content. ADAPT = Aim to Decrease Anxiety and Pain Treatment; N/A = item was not included in the module.

**Table 3:** Effectiveness of Skills.

**Effective (*n, %)***	**Prompt**	**Participant Response**
Problem Solving (*n* = 20/48, 41.7% completed)		
Effective (20/20, 100%)	Identify your problem:	I get extreme belly pain when I swim causing me to want to sit out with my friends
	Identify your thoughts, feelings, and behavior:	Thought: My friends will be mad at me if I don’t keep playing in the pool Feelings: Worry that the pain will worsen and Sad if I choose to sit out
	List coping skills you have learned so far:	Activity pacing, Progressive muscle relaxation, Positive statements, Mini relaxation, Deep breathing, Guided imagery
	Pick several coping skills to use to deal with this problem:	Activity Pacing, Positive Statements, Deep Breathing, Mini Relaxation
	Predict what will happen when you try the solution(s):	When I do activity pacing I think this will allow me to take some smaller breaks causing my belly to have time to stop hurting… When I do positive statements I would expect that the worry would decrease or stop …when I do deep breathing and mini relaxation I would expect to clear some of the negative thoughts and focus more on the swimming and fun than my pain
	What actually did happen when you tried the solution(s):	the activity pacing extremely helped with the pain to the point there almost was no pain the positive statements reminded me that I had gotten though the same pain before so it was not going to control me this time The deep breathing helped because it feels refreshing to my stomach to take a deep breath and release almost what feels like tension The mini relaxation reminded me that I was trying to have fun and would distract me from the pain
	What, if anything would you do differently next time:	try to stay in the pool longer and try guided imagery on the pool float
**Effective (*n*, *%*)**	**Prompt**	**Participant Response**
Detective Thinking (*n* = 12/48, 25.0% completed)		
Effective (10/12, 83.3%)	Event:	I am going somewhere new and don’t know where
	Thoughts:	What if I am late or way to early? Am I sure this is where I’m going? Did I wear the appropriate thing? Was I supposed to bring something?
	Worry rating before:	9 out of 10
	What is the evidence?	I think this every time and I never am late… I could ask someone and see if I am going the right way. Don’t worry about what your wearing you look fine. …Plus people are late to things all the time.
	What is a realistic thought?	You won’t be late and even if you are its not like it’ll be a big deal.
	New worry rating:	6 out of 10

Note. Effectiveness of skills was determined by participants’ indication of how helpful skills were to manage pain (problem solving) or anxiety (detective thinking). The examples above also demonstrate *appropriate use* of skills. Specifically, the problem solving example denotes an overall appropriate use of the skill, though only a thought and feeling are labeled in response to identifying thoughts, feelings, and behaviors.

## Data Availability

The data presented in this study are available upon request from the corresponding author.
